# The interaction of HAb18G/CD147 with integrin α6β1 and its implications for the invasion potential of human hepatoma cells

**DOI:** 10.1186/1471-2407-9-337

**Published:** 2009-09-23

**Authors:** Jing-yao Dai, Ke-feng Dou, Cong-hua Wang, Pu Zhao, Wayne Bond Lau, Ling Tao, Ya-mei Wu, Juan Tang, Jian-li Jiang, Zhi-nan Chen

**Affiliations:** 1 Cell Engineering Research Centre & Department of Cell Biology, State Key Laboratory of Cancer Biology, Fourth Military Medical University, No.17 West Changle Road, Xi'an 710032, Shaanxi, PR China; 2 Department of Hepatobiliary Surgery, First Affiliated Hospital, Fourth Military Medical University, No.17 West Changle Road, Xi'an 710032, Shaanxi, PR China; 3 Department of Clinical Immunology, First Affiliated Hospital, Fourth Military Medical University, No.17 West Changle Road, Xi'an 710032, Shaanxi, PR China; 4 Department of Emergency Medicine, Thomas Jefferson University, 1015 Walnut Street, Philadelphia, PA, 19107, USA; 5 Department of Cardiology, First Affiliated Hospital, Fourth Military Medical University, No.17 West Changle Road, Xi'an 710032, Shaanxi, PR China

## Abstract

**Background:**

HAb18G/CD147 plays pivotal roles in invasion by hepatoma cells, but the underlying mechanism remains unclear. Our previous study demonstrated that overexpression of HAb18G/CD147 promotes invasion by interacting with integrin α3β1. However, it has never been investigated whether α3β1 is solely responsible for this process or if other integrin family members also interact with HAb18G/CD147 in human hepatoma cells.

**Methods:**

Human SMMC-7721 and FHCC98 cells were cultured and transfected with siRNA fragments against HAb18G/CD147. The expression levels of HAb18G/CD147 and integrin α6β1 were determined by immunofluorescent double-staining and confocal imaging analysis. Co-immunoprecipitation and Western blot analyses were performed to examine the native conformations of HAb18G/CD147 and integrin α6β1. Invasion potential was evaluated with an invasion assay and gelatin zymography.

**Results:**

We found that integrin α6β1 co-localizes and interacts with HAb18G/CD147 in human hepatoma cells. The enhancing effects of HAb18G/CD147 on invasion capacity and secretion of matrix metalloproteinases (MMPs) were partially blocked by integrin α6β1 antibodies (*P *< 0.01). Wortmannin, a specific phosphatidylinositol kinase (PI3K) inhibitor that reverses the effect of HAb18G/CD147 on the regulation of intracellular Ca^2+ ^mobilization, significantly reduced cell invasion potential and secretion of MMPs in human hepatoma cells (*P *< 0.05). Importantly, no additive effect between Wortmannin and α6β1 antibodies was observed, indicating that α6β1 and PI3K transmit the signal in an upstream-downstream relationship.

**Conclusion:**

These results suggest that α6β1 interacts with HAb18G/CD147 to mediate tumor invasion and metastatic processes through the PI3K pathway.

## Background

CD147 is a transmembrane glycoprotein categorized as a member of the immunoglobulin superfamily (IgSF) [1-4]. CD147 was identified independently in various species and referenced throughout the literature as EMMPRIN (Extracellular Matrix Metalloproteinase-Inducer), M6 and HAb18G (human) [1,2,5], Neurothelin, 5A11 and HT7 (chicken) [6-8], OX47 and CE9 (rat) [3,9], and Basigin, gp42 (human and mouse) [4,10]. CD147 plays pivotal roles in the intercellular interactions involved in tumor metastasis and angiogenesis, spermatogenesis and fertilization [11,12], neural network formation and development [13,14], HIV infection, and rheumatoid arthritis [15,16]. Most importantly, studies from other investigators and our own laboratory have demonstrated that CD147 acts as a central factor in the stimulation of matrix metalloproteinases (MMPs) and promotes tumor invasion. However, intracellular signaling mechanisms responsible for CD147's stimulation of MMPs and tumor promoting effects remain incompletely understood.

Integrins are cell surface adhesive receptors composed of α- and β-chain heterocomplexes that mediate physical and functional interactions between cells and the extracellular matrix. Variant integrins can interact with different ligands and vice versa. Integrins thus serve as bidirectional transducers of extracellular and intracellular signals in the processes of cell adhesion, proliferation, differentiation, apoptosis, and tumor progression. Normal adult hepatocytes express low levels of only three integrins: α1β1 (a collagen and laminin receptor), α5β1 (a fibronectin receptor), and α9β1 (a tenascin receptor). In contrast, other integrins (such as α2β1, α3β1, α6β1, and α6β4) are not present in normal hepatocytes, but are expressed in hepatoma cells. However, the precise roles integrins play in liver carcinogenesis remain unclear.

In previous studies, CD147 was found to be associated with integrins α3β1 and α6β1, but not α2β1 and α5β1 [17]. In a recent study, we demonstrated that α3β1 plays a critical role in CD147-mediated liver carcinogenesis, indicating that the interaction between CD147 and various integrins is a necessary step for their tumor-promoting effects [18]. However, it is unknown whether α3β1 is solely responsible for this process or if other integrin family members also interact with HAb18G/CD147 in human hepatoma cells. Given the fact that one of the most frequent alterations during liver carcinogenesis is de novo expression of the integrin α6β1 and that the induction of α6β1 expression is an early event in hepatocellular carcinogenesis [19-23], it is critical to clarify whether α6β1 interacts with CD147 and thus contributes to liver carcinogenesis.

In the present study, we demonstrated that HAb18G/CD147 interacts with integrin α6β1, activates the PI3K signal pathway through phosphorylation, and thereby enhances the invasion potential of hepatoma cells.

## Methods

### Cell culture

Human SMMC-7721 and FHCC98 cells (both obtained from the Institute of Cell Biology, Academic Sinica, Shanghai, China) were cultured with RPMI 1640 medium (Gibco, New York, USA) supplemented with 10% fetal bovine serum (FBS, Gibco, New York, USA), 1% penicillin/streptomycin, and 2% L-glutamine at 37°C in a humidified atmosphere of 5% CO_2_.

### Gene silencing of CD147 by RNA interference, RT-PCR and Western blot

The sense sequence for the HAb18G/CD147 small interfering RNA (siRNA) was 5'-GUU CUU CGU GAG UUC CUC TT-3', 3'-DTD TCA AGA AGC ACU CAA GGA G-5' (Ambion, USA). FHCC98 and 7721 cells were transfected with siRNA using Lipofectamine 2000 reagent (Invitrogen, USA) according to the manufacturer's instructions. Silencer-negative control siRNA (sncRNA) (Ambion, USA) was used as a negative control under similar conditions. Silencing effects of HAb18G/CD147 were examined by RT-PCR and Western blot.

Forty-eight hours after siRNA transfection, total RNA was isolated using Trizol (Invitrogen, Carlsbad, CA, USA) according to the manufacturer's instructions for use in the analysis of CD147 mRNA levels. The isolation was followed by first-strand cDNA synthesis using True Script reverse transcriptase (BioRad, Hercules, CA, USA). The cDNA was amplified by PCR using a specific primer set for CD147 or glyceraldehyde phosphate dehydrogenase (GAPDH) as an internal control. The sequences of the upstream and downstream primers were, respectively, as follows:

Sense: 5'-ACA TCA ACG AGG GGG AGA CG-3'; Antisense: 5'-GGC TTC AGA CAG GCA GGA CA-3' for CD147 (492 bp); Sense: 5'-CTG AAC GGG AAG CTC ACT GG-3'; Antisense: 5'-TGA GGT CCA CCA CCC TGT TG-3' for GAPDH (313 bp). PCR analysis was performed under the following conditions: an initial denaturation at 94°C for 2 min followed by 25-30 cycles of a 30-sec denaturation step at 94°C, renaturation for 60 sec at 57°C, and extension for 90 sec at 72°C. The amplified products were analyzed by 1% agarose gel electrophoresis followed by GoldView™ staining. Forty-eight hours after siRNA transfection, FHCC98 and 7721 cells were harvested in a lysis buffer and equal amounts of cellular proteins were subjected to SDS-PAGE (10%). Proteins were transferred to polyvinylidene difluoride membranes and blots were probed with HAb18 mAb (prepared in our laboratory). GAPDH was chosen as an internal control and the blots were probed with mouse anti-GAPDH mAb (Chemicon International, Inc.).

### Co-immunoprecipitation and Western blot analyses

The interaction of HAb18G/CD147 with integrin α6β1 in native cells was detected using the ProFound™ Mammalian Co-Immunoprecipitation Kit (Pierce, USA) according to the manufacturer's instructions. Briefly, FHCC98 cells or 7721 cells (1×10^6^) were lysed with M-per reagent. The lysate was collected onto a coupling gel by four washes with the co-immunoprecipitation buffer and which were pre-bound with 200 μg mouse anti-human HAb18G/CD147 mAb/HAb18 (developed previously in our lab [24-27]) or 200 μg mouse anti-human α6 mAb (GoH3) (Santa Cruz, USA) and 200 μg mouse anti-human β1 mAb (3S3) (Santa Cruz, USA). Bound proteins were eluted from the coupling gel with elution buffer, and aliquots of the elution were analyzed by Western blot using goat anti-human α6 mAb (diluted 1:2000), anti-human β1 antibody (diluted 1:2000) or HAb18 (diluted 1:5000). Horseradish peroxidase (HRP)-conjugated rabbit anti-goat IgG (diluted 1:5000; Amersham Pharmacia) was used as the negative control.

### Immunofluorescence and confocal microscopy

Cells were allowed to attach to precoated glass coverslips overnight. They were fixed the following day in 4% paraformaldehyde and then blocked with 2% bovine serum albumin (BSA) in phosphate-buffered saline (PBS) for 0.5 h. Coverslips were incubated with the primary antibodies (Santa Cruz) at a 1:200 dilution in PBS for 1 h. Primary antibody-treated cells were washed in PBS and then incubated with Allex594 goat anti-mouse (Invitrogen-Molecular Probe, USA) or fluorescein isothiocyanate (FITC)-conjugated donkey anti-goat secondary antibodies (Santa Cruz, USA) at a 1:500 dilution in PBS for 1 h. Cell nuclei were dyed with 4,6-diamino-2-phenyl indole (DAPI) (Invitrogen-Molecular Probe) for 3 min. Finally, the cells were mounted using glycerol and observed by FV1000 laser scanning confocal microscope (Olympus).

### Invasion assay

The assay was performed using chambers with polycarbonate filters with 8-μm nominal pore size (Millipore, USA) coated on the upper side with Matrigel (Becton Dickinson Labware, USA). The chambers were placed into a 24-well plate. Hepatoma cells were harvested after 24 h of siRNA transfection. Five groups of transfected cells (1×10^5^) were harvested and placed in the upper chamber: transfected cells alone, cells treated with 10 μg/mL anti-integrin α6β1 mAb, cells treated with 1 μg/mL Wortmannin in 100 μL RPMI 1640 containing 0.1% BSA, sncRNA-transfected cells (negative control), and untreated cells (blank control). The lower chamber was filled with 600 μL RPMI 1640 containing 5% FBS and then incubated for 24 h at 37°C in a humidified atmosphere containing 5% CO_2_. Cells remaining in the upper chamber were completely removed by gently swabbing. The number of cells that passed through the filter and invaded the lower chamber was determined using a colorimetric crystal violet assay.

### Gelatin zymography

Twenty-four hours after siRNA transfection, cells were treated with antibodies or inhibitors in serum-free medium. Cells were incubated at 37°C for 20 h. The conditioned medium was collected and separated by 8% acrylamide gels containing 0.1% gelatin. The gels were incubated in a 2.5% Triton X-100 (Sigma, USA) solution at room temperature with gentle agitation and then were soaked in reaction buffer [50 mM Tris-HCl (pH 7.5), 200 mM NaCl, and 10 mM CaCl_2_] at 37°C overnight. The gels were stained for 6 h and destained for 0.5 h. The zones of gelatinolytic activity were revealed by negative staining.

### Western blot for Akt phosphorylation

FHCC98 and 7721 cells were harvested in a lysis buffer, and equal amounts of cellular proteins were subjected to SDS-PAGE (10%). Proteins were transferred to PVDF membranes, and blots were probed with phosphorylated and non-phosphorylated forms of Akt. Blots were washed 3-5 min with Tris-buffered saline/0.1% Tween 20 and incubated with horseradish peroxidase-conjugated secondary antibody (1:2000) for 1 h. The membranes were washed as described above, and the bands detected by chemilluminescence (Amersham, Freiburg, German).

### Statistical analysis

Statistical significance was determined using a one-way ANOVA analysis or Student's t-test. GraphPad Prism software (Cricket Software, Philadelphia, PA) was used for the above analyses and *P *values less than 0.05 were considered significant.

## Results

### HAb18G/CD147 co-localized with α6β1 integrin on the surface of human hepatoma cells

**Figure 1:**
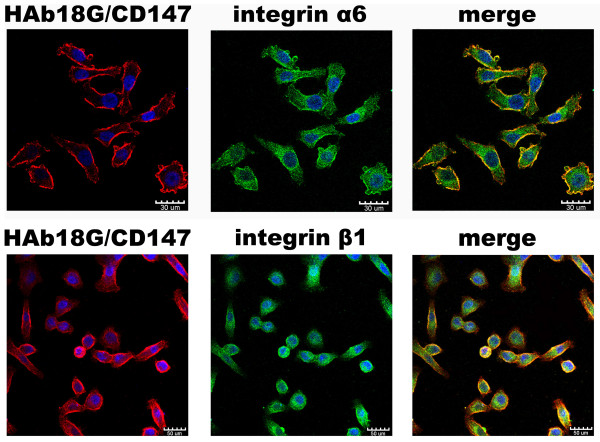
**Expression and co-localization of HAb18G/CD147 with integrin α6 and β1 subunits in FHCC98 cells**. FHCC98 cells were double-stained for HAb18G/CD147 (red) and integrin α6 or β1 (green).

Immunofluorescent double-staining and confocal imaging analysis demonstrated that integrin α6β1 and HAb18G/CD147 were strongly expressed at the marginal areas of the two different hepatoma cell lines FHCC98 (Fig. 1) and 7721 (not shown). HAb18G/CD147 signals co-localized with integrin α6 and β1 on the surface of HCC cells. The co-localizations were distributed diffusely throughout the membrane.

### HAb18G/CD147 immunoprecipitates with α6β1 integrin in human hepatoma cells

**Figure 2:**
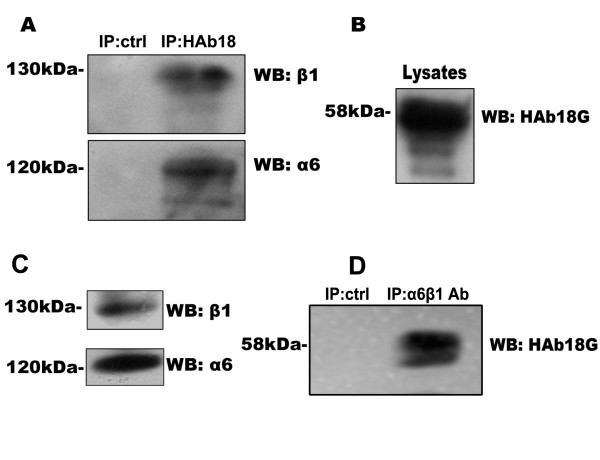
**Immunoprecipitation of HAb18G/CD147 and integrin α6 and β1 subunits in FHCC98 cells**. (A) Precipitates from HAb18G/CD147 immunocomplexes were assayed for precipitated integrin α6 and β1 subunits. Precipitates from irrelevant antibody (Mouse IgG) were used as a negative control. (B) Expression of HAb18G/CD147 in FHCC98 cell lysates by Western blot. (C) Expression of integrin α6 and β1 subunits in FHCC98 cell lysates by Western blot. (D) Precipitates from α6β1 immunocomplexes were assayed for precipitated HAb18G/CD147. Precipitates from irrelevant antibody (Mouse IgG) were used as a negative control.

To verify whether HAb18G/CD147 interacts with integrin α6β1 in hepatoma cells, we performed co-immunoprecipitation experiments in FHCC98 (Fig. 2) and 7721 (not shown) cells. As Fig. 2 demonstrates, integrin α6 and β1 subunits co-immunoprecipitate with endogenous HAb18G/CD147 in FHCC98 cells. The same finding occurred in 7721 cell lysates (results not shown). The results indicated that HAb18G/CD147 and α6β1 integrin interact in their native conformations.

### Effects of silencing HAb18G/CD147 on FHCC98 and 7721 cells

**Figure 3:**
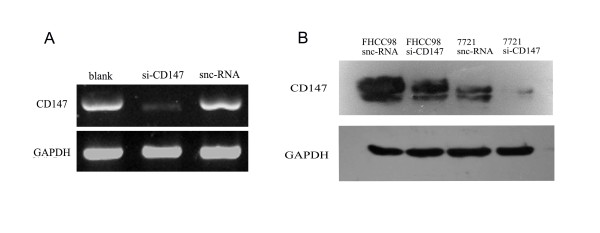
**Effect of silencing HAb18G/CD147 in FHCC98 and 7721 cells**. Forty-eight hours after siCD147 or sncRNA transfection of FHCC98 and 7721 cells, HAb18G/CD147 expression levels were examined by RT-PCR (A) and Western blot (B).

To investigate the role of HAb18G/CD147 in FHCC98 and 7721 cells, RNA interference was used to knock down the expression of HAb18G/CD147 in these two cell lines. The HAb18G/CD147-specific siRNA (siCD147) and sncRNA were tested for their ability to specifically suppress HAb18G/CD147. RT-PCR showed that the sncRNA was incapable of inhibiting HAb18G/CD147 gene expression, whereas siCD147 could effectively decrease the mRNA expression of HAb18G/CD147 (Fig. 3A). Similar results were obtained in 7721 cells (data not shown). These results were confirmed by Western blot. The protein expression of HAb18G/CD147 was obviously decreased in siCD147-transfected cells, but not in sncRNA-transfected cells 48 h after siRNA transfection (Fig. 3B). These data show that siCD147 treatment effectively decreased HAb18G/CD147 expression in FHCC98 and 7721 cells.

### Integrin α6β1 mediates HAb18G/CD147-induced invasion of human hepatoma cells

**Figure 4:**
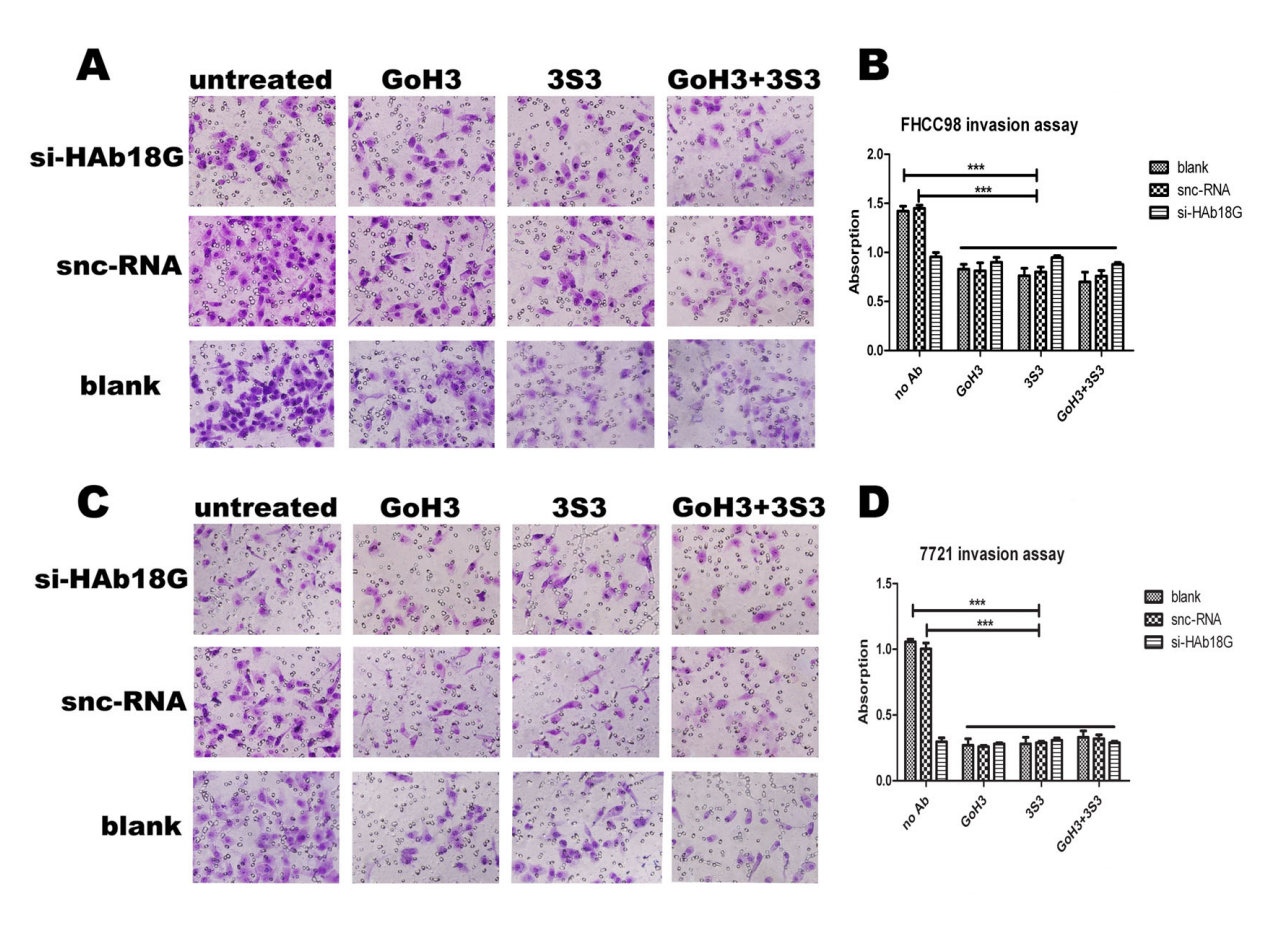
**Invasion of hepatoma cells with or without integrin α6β1 mAbs and siHAb18G/CD147 treatment**. (A-D) Matrigel invasion assay of FHCC98 (A, B) and 7721 (C, D) cells. Bars represent the mean of triplicate samples; error bars represent standard deviation. Data are representative of three independent experiments. ***P < 0.01 versus corresponding cells with no antibody treatment.

In order to clarify the functional association of HAb18G/CD147 with integrin α6β1, the effect of anti-integrin α6 mAb (GoH3) and β1 mAb (3S3) on the invasion of FHCC98 and 7721 cells were examined in the presence of HAb18G/CD147 gene silencing. Silencing of the HAb18G/CD147 gene was achieved by transfection of FHCC98 and 7721 cells with the HAb18G/CD147-specific siRNA. To explore the nature of the integrins involved in HAb18G/CD147-mediated invasion, experiments were reproduced with blocking antibodies to α6 and β1. As shown in Fig. 4, there was no significant difference between the invasive cell numbers of sncRNA-transfected and blank FHCC98 cells. In contrast, transfection with sncRNA+GoH3, sncRNA+3S3, and sncRNA+GoH3+3S3 reduced invasive cell numbers by 32.37 ± 14.10%, 31.07 ± 4.82%, and 34.89 ± 12.15%, respectively, as compared to the blank group (*P *< 0.01). FHCC98 cells transfected with siHAb18G/CD147 demonstrated significantly reduced invasive cell numbers by 28.01 ± 5.56% (*P *< 0.01), but there was no significant difference in comparison to sncRNA+GoH3, sncRNA+3S3, and sncRNA+GoH3+3S3 cell groups. When cells were treated with siHAb18G/CD147+GoH3, si-HAb18G/CD147+3S3, or siHAb18G/CD147+GoH3+3S3, the invasive cell numbers were also significantly reduced by 29.21 ± 9.18%, 23.53 ± 6.44%, and 30.28 ± 8.04%, as compared to the blank group respectively (*P *< 0.01). In comparison to the siHAb18G/CD147-treated alone group, no difference was found. Similar results were obtained in 7721 cells (data not shown).

**Figure 5:**
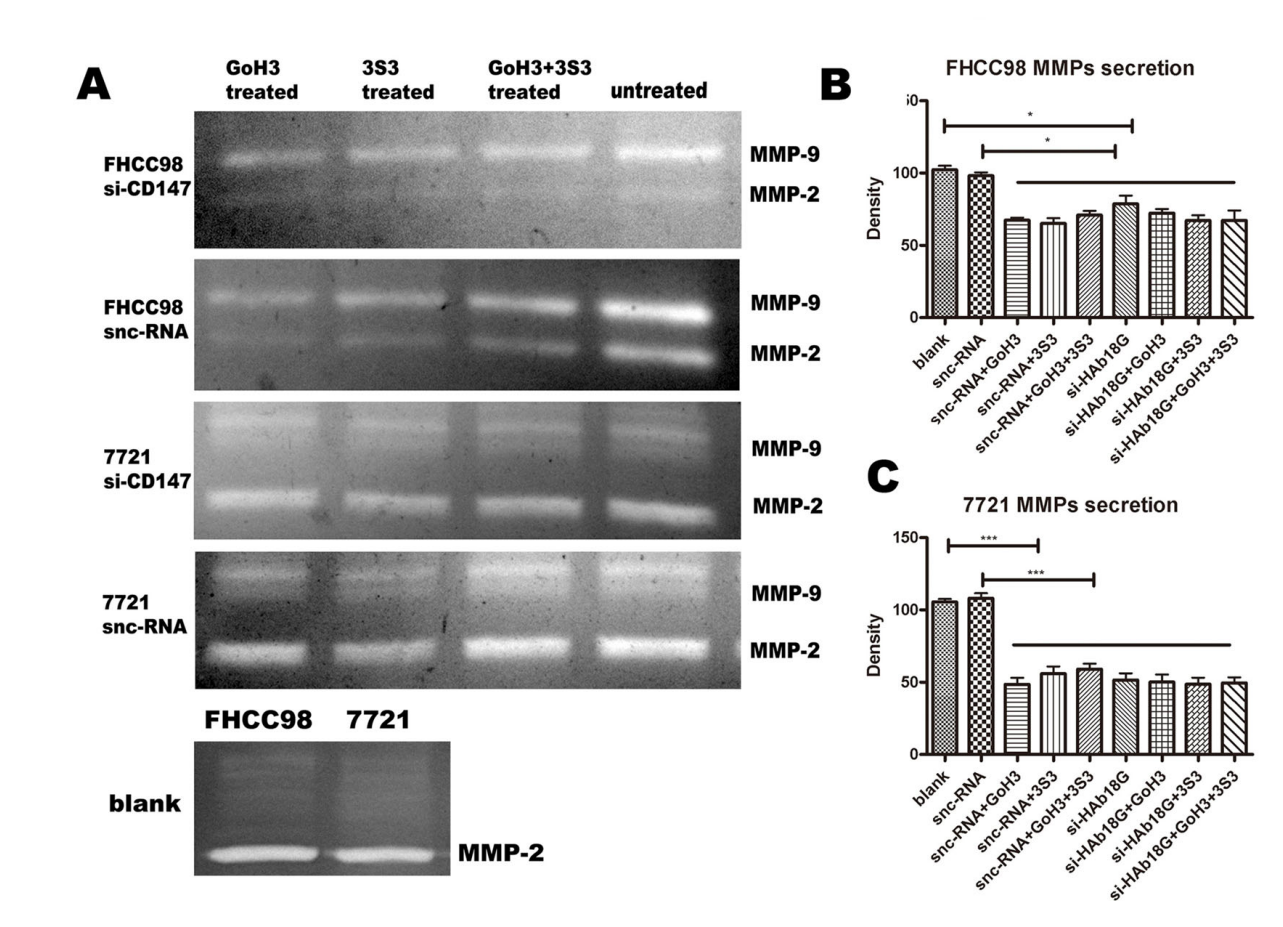
**MMP secretion of hepatoma cells with or without integrin α6β1 mAbs and siHAb18G/CD147 treatment**. MMP levels of FHCC98 (A, B) and 7721 (A, C) cells. Bars represent the mean of triplicate samples; error bars represent standard deviation. Data are representative of three independent experiments. ***P < 0.01, * *P *< 0.05 versus corresponding cells with no antibody treatment.

After siRNA transfection or treatment with blocking mAb against integrin α6β1, the supernatants of FHCC98 and 7721 cells were collected to test the levels of MMP secretion. Results from the gelatin zymography assay showed that secretion of both MMP-2 and MMP-9 was significantly reduced in FHCC98 and 7721 cells after treatment with siRNA or antibodies. As shown in Fig. 5, the density of MMPs in FHCC98 cell lines after treatment with sncRNA associated with GoH3, 3S3, and GoH3+3S3 was reduced by 32.55 ± 3.01%, 34.81 ± 6.25%, and 29.09 ± 5.33%, respectively (*P *< 0.01), as compared to the blank group. The MMPs levels in siHAb18G/CD147-transfected FHCC98 cells were also significantly reduced by 21.31 ± 9.98% (*P *< 0.01), but there was no significant difference in comparison to the sncRNA+GoH3, sncRNA+3S3, and sncRNA+GoH3+3S3 groups. The density of MMPs in the siHAb18G/CD147+GoH3, siHAb18G/CD147+3S3, and siHAb18G/CD147+GoH3+3S3 groups were also significantly reduced by 27.75 ± 5.03%, 32.78 ± 6.30%, and 32.63 ± 11.90% (*P *< 0.05), respectively, as compared to the blank group, but no significant difference was found in comparison to the siHAb18G/CD147-transfected cells alone. Similar results were obtained in 7721 cells (data not shown). The results of the invasion assay and gelatin zymography suggested that there were no additive effects between HAb18G/CD147 gene silencing and α6β1 antibodies.

### PI3K is involved in HAb18G/CD147-elevated invasion of human hepatoma cells

**Figure 6:**
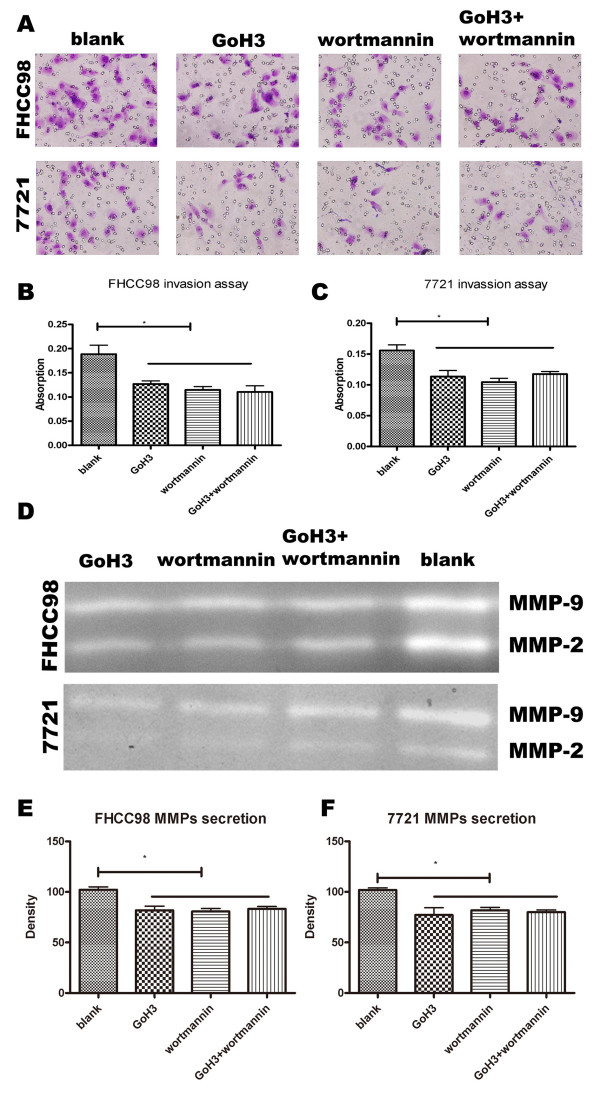
**Effects of a PI3K inhibitor on invasion and MMP secretion of FHCC98 and 7721 cells**. (A-C) Matrigel invasion assay of FHCC98 (A, B) and 7721 (A, C) cells. (D-F) MMP levels of FHCC98 (D, E) and 7721 (D, F) cells. Bars represent the mean of triplicate samples; error bars represent standard deviation. Data are representative of three independent experiments. * *P *< 0.05 versus corresponding untreated cells.

We further tested the role of PI3K on HAb18G/CD147-elevated invasion potential in order to identify the downstream signal molecule of the HAb18G/CD147-induced integrin-involved pathway. Wortmannin, an irreversible inhibitor of PI3K, was introduced into the invasion and zymography assays. As shown in Fig. 6, exposure to Wortmannin for 40 min inhibited invasion and MMP secretion. In FHCC98 cells, when treated with anti-α6 mAb, Wortmannin, and associated anti-α6 mAb, the number of invasive cells were reduced by 32.69 ± 3.06%, 39.22 ± 2.31%, and 41.52 ± 2.33%, respectively, as compared to the blank control group (*P *< 0.05) (Fig. 6A and 6B). In 7721 cells, the invasive cell numbers were correspondingly reduced by 27.19 ± 2.08%, 32.98 ± 4.73%, and 24.63 ± 1.69% (*P *< 0.05) (Fig. 6A and 6C). There were no significant differences among anti-α6 mAb, Wortmannin, and Wortmannin-associated anti-α6 mAb.

**Figure 7:**
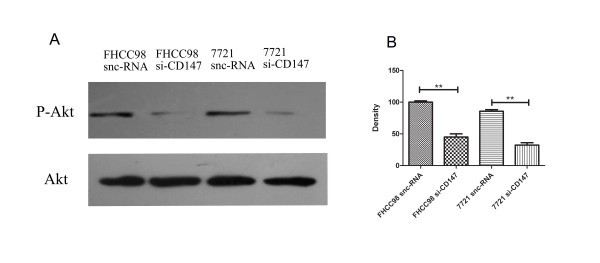
**Akt phosphorylation in FHCC98 and 7721 cells**. Forty-eight hours after siCD147 or sncRNA transfection of FHCC98 and 7721 cells, phospho-Akt and total-Akt expression levels were examined by Western blot. Bars represent the mean of triplicate samples; error bars represent standard deviation. Data are representative of three independent experiments. ** *P *< 0.01 versus corresponding controls.

The results of gelatin zymography also showed that MMP secretion was significantly reduced in FHCC98 and 7721 cells after treatment with Wortmannin and anti-α6 mAb. The density of MMPs in the anti-α6 mAb, Wortmannin, and Wortmannin-associated anti-α6 mAb was reduced by 18.82 ± 7.27%, 19.26 ± 5.21%, and 16.85 ± 4.13%, respectively, as compared to the blank control group in FHCC98 cells (*P *< 0.05) (Fig. 6D and 6E). In 7721 cells, these same groups correspondingly manifested reduced MMP density by 22.82 ± 12.32%, 18.18 ± 4.97%, and 20.03 ± 3.74% (*P *< 0.05) (Fig. 6D and 6F). There were no significant differences among groups treated with anti-α6 mAb, Wortmannin, or anti-α6 mAb associated with Wortmannin (*P *> 0.05). To identify whether Akt phosphorylation is involved in the HAb18G/CD147-mediated and integrin-activated invasion processes of human hepatoma cells, the expression levels of Akt and P-Akt were tested by Western blot. The expression levels of P-Akt decreased 57.62 ± 3.61% and 68.06 ± 4.43%, respectively, in siCD147-transfected FHCC98 cells and siCD147-transfected 7721 cells when compared to expression levels in sncRNA-transfected cells (*P *< 0.01, Fig. 7). All these results suggest that PI3K, a key downstream signal molecule of integrin α6β1, is involved in HAb18G/CD147-induced invasion and metastatic processes of human hepatoma cells.

## Discussion

The invasion process is a complicated and fatal cascade process in the human hepatoma, but the underlying molecular events remain largely unknown. Our previous studies implicated a hepatoma-associated antigen, HAb18G/CD147, in metastatic processes [28-30]. However, ligands or receptors for HAb18G/CD147 remain obscure, and whether HAb18G/CD147 directly interacts with extra- or intracellular molecules to execute its function is still unknown. We report here that HAb18G/CD147, by interacting with α6β1 integrin, enhances the metastatic potential of hepatoma cells.

In this study, we identified previously uncharacterized roles for HAb18G/CD147. The expression of integrin α6β1, a receptor of laminin, was positively related to the expression of HAb18G/CD147 as indicated the immunofluorescent double-staining and co-immunoprecipitation assay. When the specific antibodies for α 6 and β1 were added to the culture system, effective blocking of the interaction of HAb18G/CD147 and integrin α6β1 reduced cell invasion and MMP secretion. In siHAb18G-transfected cells, the addition of specific α6 and β1 antibodies provides no significant additive inhibitory effect. These results suggest that the enhancing effect of HAb18G/CD147 on cell invasion and metastatic potential is mediated through α6β1 integrin.

Integrins serve as the major receptors mediating cell-matrix adhesion, which plays an important role in the invasive process of tumor cells. Therefore, it is not surprising that their interaction with laminin, the major component of the basement membrane, serves as a key event in the tumor invasion and metastatic process [31]. Through the interaction with integrins, CD147 may regulate the integrin-laminin association and then influence diverse processes such as basement membrane formation. The interaction of CD147 with integrin is conserved in Drosophila and is proposed to play a role in dorsal closure and extraembryonic membrane apposition [32] as well as in the maintenance of cellular architecture through cytoskeletal rearrangement [18]. Within cultured insect cells, the CD147-integrin interaction is essential for lamellipodia formation. Within retinal cells, disruption of the CD147-integrin interaction results in aberrant organelle distribution, including mitochondria, nuclei, and rough endoplasmic reticulum [18]. The elevated expression levels of both CD147 and integrin α6β1 have been observed in most metastatic and primary cancers [33,34]. Comparison of normal prostate cells to cancerous cells showed a preferential pairing of α6 with β4 transitioning to α6 with β1 in cancer cells [35]. The significance of this transition, while still speculative, is most likely related to alterations in downstream signaling events. But there are no reports about the signal mechanism of the CD147-integrin/α6β1 interaction.

The signaling pathways of the integrins consist of many cytoskeleton proteins and enzymes, of which focal adhesion kinase (FAK), paxillin, and PI3K are crucial components. We previously discovered that FAK, paxillin, and their phosphorylation levels closely correlate with HAb18G/CD147 expression in human hepatoma cells [30]. In the present study, a specific inhibitor of PI3K, a key protein kinase downstream of integrin α6β1, significantly blocked HAb18G/CD147-induced invasion and MMP release. Our previous data have already demonstrated that elevated HAb18G/CD147 expression leads to attenuation of the store-operated Ca^2+ ^entry response to NO/cGMP and enhanced metastatic potential [26], but the precise mechanism is still unknown. As a strong inducer for intracellular Ca^2+ ^store release, IP3 is stimulated by PI3K and activates the Ca^2+ ^channels, allowing greater Ca^2+ ^influx into the cells. We may speculate that HAb18G/CD147 enhances the invasion and metastatic potential of human hepatoma cells via integrin α6β1-PI3K-Ca^2+ ^signaling pathways.

## Conclusion

In the present study, we have identified the interaction of HAb18G/CD147 with integrin α6β1 in human hepatoma cells. We demonstrated that HAb18G/CD147 promotes invasion potential of hepatoma cells by interacting with integrin α6β1 and further activating its downstream PI3K-Akt signaling pathway. These findings shed new light onto the mechanisms underlying HAb18G/CD147-induced invasion and human hepatoma cell metastatic processes. The CD147-integrin α6β1 interaction might be a novel potential target for tumor metastasis therapy.

## Competing interests

The authors declare that they have no competing interests.

## Authors' contributions

JYD, KFD performed the studies and drafted the manuscript. CHW participated in the statistical analysis and helped to draft the manuscript. ZNC, JLJ participated in the design of the study. Wayne Bond Lau, LT participated in the writing assistance. JT, YMW, PZ provided purely technical help. All authors have read and approved the final manuscript.

## Pre-publication history

The pre-publication history for this paper can be accessed here:

http://www.biomedcentral.com/1471-2407/9/337/prepub
